# Neural Signature of DCD: A Critical Review of MRI Neuroimaging Studies

**DOI:** 10.3389/fneur.2016.00227

**Published:** 2016-12-16

**Authors:** Maëlle Biotteau, Yves Chaix, Mélody Blais, Jessica Tallet, Patrice Péran, Jean-Michel Albaret

**Affiliations:** ^1^URI Octogone-Lordat (EA 4156), Université Toulouse II Jean Jaurès, Toulouse, France; ^2^Toulouse NeuroImaging Center, INSERM, University of Toulouse Paul Sabatier, Toulouse, France; ^3^Children’s Hospital, Toulouse-Purpan University Hospital, Toulouse, France

**Keywords:** neurodevelopmental disorder, neuroimaging, brain, developmental coordination disorder, neural correlates

## Abstract

The most common neurodevelopmental disorders (e.g., developmental dyslexia (DD), autism, attention-deficit hyperactivity disorder (ADHD)) have been the subject of numerous neuroimaging studies, leading to certain brain regions being identified as neural correlates of these conditions, referring to a neural signature of disorders. Developmental coordination disorder (DCD), however, remains one of the least understood and studied neurodevelopmental disorders. Given the acknowledged link between motor difficulties and brain features, it is surprising that so few research studies have systematically explored the brains of children with DCD. The aim of the present review was to ascertain whether it is currently possible to identify a neural signature for DCD, based on the 14 magnetic resonance imaging neuroimaging studies that have been conducted in DCD to date. Our results indicate that several brain areas are unquestionably linked to DCD: cerebellum, basal ganglia, parietal lobe, and parts of the frontal lobe (medial orbitofrontal cortex and dorsolateral prefrontal cortex). However, research has been too sparse and studies have suffered from several limitations that constitute a serious obstacle to address the question of a well-established neural signature for DCD.

## Introduction

Developmental coordination disorder (DCD) is a highly prevalent neurodevelopmental disorder (1.8–6% of school-aged children), with an early age of onset and persistence into adulthood (1–3). Although it is a heterogeneous disorder (4) with different phenotypes (5), it can be characterized by a persistent motor impairment that negatively affects daily living activities and/or academic achievement and cannot better be explained by intellectual disability or an underlying neurological condition (1). Posture, motor learning, and sensorimotor coordination are the main areas of functional difficulties experienced by children with DCD (6). The motor performances of children with DCD are slower, less accurate, and more variable than those of their peers. Characteristic signs include poor distal control and hand coordination, impaired balance, and difficulty in motor learning and motor imagery (5, 7–10), which, in turn, cause numerous functional difficulties [e.g., dressing, writing, using utensils, running, catching balls, and playing sports (11)].

No single cause has been identified, and its etiology appears to be multifactorial (12, 13). However, for many years, brain features have been assumed to constitute a valid explanation for all the symptoms in DCD, and several hypotheses have been formulated accordingly, some substantiated by behavioral studies.

It is, therefore, worth wondering whether it is currently possible to identify a neural signature for DCD, i.e., define the neuroanatomic basis of DCD (one or more brain areas or brain networks) in relationship with symptoms of disorder. For this purpose, we first reviewed studies that have made assumptions about specific brain areas linked to DCD, and secondly, all magnetic resonance imaging (MRI) studies realized in DCD population.

## Neural Correlates of DCD: From Early Hypotheses to the First Behavioral Studies

There have been three critical stages in the search for a link between brain characteristics and the symptoms of DCD: (i) assumptions based on brain lesions (studies investigating the behavior of individuals with brain damage); (ii) brain hypotheses based on behavioral data (inferences drawn from neurological examinations about which neural circuitries or brain areas are involved); and (iii) neuroimaging studies, especially MRI. In this section, we deal with the first two stages.

### Early Assumptions about the Brain

The clinical symptoms (difficulties in postural control, balance, coordination, motor planning, or learning) of an underlying brain impairment were first reported in the 1960s, leading to several different assumptions about the relevant brain features, including impaired brain dominance (14) and hemispheric disconnection (15). The presence of soft neurological signs (16) also suggested an atypical recruitment of the cerebellum, basal ganglia (BG), and parietal and frontal lobes (17–19). For example, Luria (20) analyzed the cerebral organization of perception and action (as well as attention, memory, speech, and intellectual processes), and discussed the role of the BG, and parietal and frontal cortices in motor dysfunctions. Observing that lesions in these areas could generate motor deficits in adults, he extended these causal links to clumsy children. He, therefore, deduced that occipitoparietal lesions could generate a visuospatial deficit, damage to the BG, and premotor cortex could impact the sequencing of elementary movements, and frontal lesions could affect action control (planning and realization). Shortly afterward, Gubbay (18) reported that clumsiness could be due to damaged neurological functions affecting the execution of movements (pyramidal and extrapyramidal circuits) and motor praxis (central nervous system, cerebellum, and peripheral nervous system). Finally, noting that underdevelopment of the cerebellum in premature or injured children causes clumsiness and difficulties in motor control, as well as in the performance of visuomotor and graphomotor tasks (despite preserved verbal skills), Lesný (19) suggested that atypical cerebellar development is implicated in DCD.

Concurrently, with these first assumptions, several more global approaches were proposed. Although they do not concern any specific disorder, they can be used to address the full range of neurodevelopmental disorders and account for the high rates of comorbidity among them. To initiate debate in neurological research on learning disorders and given that neurodevelopmental disorders are typically non-specific (symptoms and syndromes), heterogeneous, and frequently co-occurring, some authors proposed the term *atypical brain development* (21). This term acknowledged the overlap between these disorders and emphasized the role of the brain in their etiology. It replaced the older concept of *minimal brain dysfunction* (22), which served broadly the same purpose. More recently, we have witnessed the advent of a third term, *early symptomatic syndromes eliciting neurodevelopmental clinical examinations* [ESSENCE (23)], which refers to the various impairments displayed by children, rather than providing a specific diagnosis. It provides descriptions of impairments in multiple areas of functioning (language, motor coordination, attention, social interaction, etc.) that are associated with many neurodevelopmental disorders and accounts of co-occurrence between them. We should also mention the term *developmental brain dysfunction*, recently introduced by Moreno-De-Luca et al. (24), which includes neurodevelopmental disorders ranging from *severe* (underpinned by severe brain dysfunctions; i.e., intellectual disability and ASD) to *lighter* (underpinned by minimal brain dysfunctions; e.g., DCD, ADHD, and DD). Finally, we should also briefly describe the *neural system typography for learning difficulties* (25, 26), which contrasts general learning disabilities secondary to an impairment of the declarative learning system (i.e., intellectual developmental disorder) with neurodevelopmental disorders secondary to an impairment of the procedural learning system (i.e., ADHD, SLI, DD, and DCD), suggesting that neurodevelopmental disorders have a common impairment of the corticostriatal and/or corticocerebellar circuits.

It is important to stress that none of the abovementioned assumptions, whether they concern a single neurodevelopmental disorder or encompass all such disorders, are anything more than suppositions. The next stage was, therefore, to conduct behavioral investigations with tasks known to involve the brain areas thought to be dysfunctional.

### Neural Correlates Inferred from Neurological and Behavioral Examinations

Between 1990 and 2010, there was a huge increase in the number of studies using brain imaging techniques to explore the neural mechanisms behind motor learning. These established objective criteria for determining which tasks activate which brain areas. Consequently, over this 20-year period, researchers were able to infer hypotheses about the neural correlates of DCD from behavioral studies, thus significantly contributing to our knowledge about the neural bases of DCD. Zwicker et al. (27) and Bo and Lee (28) comprehensively reviewed the behavioral evidence for the neural correlates of DCD, exploring dominant hypotheses and their relationships with motor skill production and learning in children with DCD, leading to the identification of three main brain bases of DCD: the cerebellum, BG, and parietal lobes.

The literature on the behavioral deficits associated with DCD strongly suggests that the cerebellum (and/or its network of connections) is a logical source of the dysfunction. An increasing number of studies have therefore been undertaken to demonstrate the role of the cerebellum in uncoordinated behaviors, clumsiness, poor coordination, and postural control (29–31) – the hallmarks of DCD. Several behavioral paradigms have been designed specifically to highlight cerebellar dysfunction, including finger-to-nose touching, rapid alternating hand movements, and even motor adaptation tasks (32, 33). Given early assumptions about cerebellar dysfunctions in DCD, some studies were conducted with these specific tasks. These showed that children with DCD tend to perform more poorly than their peers on tasks such as finger-to-nose touching and rapid alternating hand movements, the most traditional tests of cerebellar function (16, 34). Difficulties with motor adaptation paradigms have also been demonstrated (35–37). Children with DCD seem to be affected by visual distortions in drawing tasks (their performance does not change significantly across trials), but seem to be able to recognize errors (adjusting their internal map) when there is an abrupt visuomotor distortion (36, 37). More recently, Lejeune et al. (38) found slower performance and lower asymptotic performance in DCD on a visuomotor adaptation task. Similar findings were reported by Cantin et al. (35, 39) and Brookes et al. (40) on a prism adaptation test, where participants with DCD were less efficient than their peers, especially for complex tasks (39). The results of these behavioral studies, therefore, point to the involvement of either the cerebellum itself, the cerebellar loops, or both in the physiopathology of DCD.

Despite growing evidence implicating the cerebellum in DCD, it is highly unlikely to be the only neural correlate. The BG also play a key role in motor control, movement initiation, movement learning, and automatization (32, 41, 42), making them a plausible source of DCD symptomatology. Lundy-Ekman et al. (16) talked about “soft neurological signs of BG dysfunction” in DCD, although their role in this disorder was essentially unknown at that time. These authors concluded that there are two coexisting subtypes of clumsiness, resulting from either BG or cerebellar dysfunction. They attributed difficulties in the timing of muscle contractions to a faulty central timer mechanism resulting from soft neurological signs of cerebellar dysfunction, and impaired control of the amplitude of the isometric force pulse to soft neurological signs of BG dysfunction. Their hypothesis was recently refined by Gheysen et al. (43) and Biotteau et al. (44), who found that children with DCD performed more poorly on the sequencing of simple movements in a serial reaction time task [SRTT; (43)] and a finger-tapping task (44) – tasks known to involve the corticostriatal network (32). It should, however, be noted that when Wilson and McKenzie (45) and Lejeune et al. (46) applied SRTT paradigms, their results did not support the BG assumption [children with DCD performed similarly to typically developing (TD) children]. Results, therefore, appear more contrasted for the BG than for the cerebellum, although there are good grounds for considering them in the neurobiology of DCD.

The final brain structure thought to be implicated in DCD is the parietal lobe. Given its role in the processing of visuospatial information, action prediction and observation, executive functions, facial recognition, and motor imagery (29, 47–50), it is legitimate to assume that it is involved in DCD. Wilson and McKenzie’s review (45) showed that children with DCD have poorer visuospatial processing than TD children. They also have difficulties with facial recognition (51), executive functions (52, 53), response inhibition (54, 55), and motor imagery (7, 10, 56, 57). All these processes involve the parietal lobes (and the prefrontal lobe for executive functions), and have, therefore, led researchers to conclude that this brain structure may be involved in DCD (7, 58, 59).

As outlined above, there is apparent evidence for the involvement of the cerebellum, BG, and parietal lobes in DCD. Some other brain areas have sporadically been identified, including the insula/claustrum, anterior cingulate cortex (ACC), dorsolateral prefrontal cortex (DLPFC), and frontal lobe (27). Some behavioral results also point to a deficit in intra- and interhemispheric transfer in DCD (55, 60). For example, Sigmundsson (60) administered behavioral tests to children with a subtype of DCD with hand–eye coordination problems to test their right- and left-hand coordination. Results indicated specific motor difficulties in controlling their non-dominant (left) hand and coordinating the left and right parts of their body, suggesting a right-hemispheric *insufficiency* (lack of hemispheric specialization) with or without a dysfunctional corpus callosum.

Taken together, these findings raise the question of whether DCD has a brain signature. Many neurodevelopmental disorders (e.g., autism, dyslexia, and ADHD) have been the subject of extensive neuroimaging research, leading to the identification of certain brain regions as neural signature of disorders. For example, left temporoparietal regions, the inferior frontal gyrus (IFG), occipitotemporal cortex, and inferior parietal lobules have been identified as neural correlates of dyslexia (61), whereas functional abnormalities in right hemispheric frontal–BG networks (related to inhibition) and the DLPFC, parietal lobes, and cerebellar areas (related to attention) are typically found in ADHD (62). So in this context, is it currently possible to identify a cerebral signature for DCD on the basis of existing neuroimaging data?

## Neuroimaging Studies

Our understanding of DCD has increased steadily over the past three decades and, as with all other neurodevelopmental disorders, it has prompted researchers to conduct neuroimaging studies. Over the past 5 years in particular, there has been a growing desire in the fields of neuroscience and psychology to understand how DCD influences neural development and functioning. Brain imaging studies have, therefore, been designed to provide evidence for the cerebral validity of DCD symptoms, based on the hypothesis that impaired perceptual motor functions are the result of atypical brain development. Nonetheless, given the acknowledged link between motor difficulties and brain features, surprisingly, few research studies have systematically explored the brains of children with DCD (Table 1). The review conducted by Peters et al. (63) highlighted this dearth of neuroimaging studies in DCD (only four MRI studies at the time). The available data were therefore extremely heterogeneous and suggested that multiple brain areas are involved in the neuropathophysiology of DCD. Two years later, Brown-Lum and Zwicker (64) listed seven MRI studies and two diffusion tensor imaging (DTI), but were unable to reach any firmer formal conclusions. Our own review, conducted 1 year later, brought five more neuroimaging studies to light. These 14 existing studies were clearly aimed at identifying the cerebral bases of the deficits observed in children with DCD, and it, therefore, seemed relevant to take a fresh look at the question of a neural correlate for DCD. Noted that we decided not to include the two MRI studies that assessed dysgraphia (65, 66), as their results, combined with those of Van Hoorn et al.’s (67) review (based on the limited data available on the neural correlate hypothesis in dysgraphia), point to the contribution of cortical areas (frontal, temporal, parietal, and occipital) and the cerebellum.

**Table 1:** **Main characteristics of the included neuroimaging studies**.

Reference	Participants	Mean age (SD); range	Gender	Inclusion and exclusion criteria	Neuroimaging acquisition	fMRI analysis	Neuroimaging results	Behavioral results
Querne et al. (68)	9 DCD 10 TD	9.9 (1.8); R = 8.0–12.9 years 10.0 (1.1); R = 8.2–11.6 years	7M, 2F 7M, 3F	DCD: DSM-IV criteria, clinical examination, parent report; low scores on NEPSY, ROCF, Stroop test; no neurological (cerebral lesion, pharmacologic medication) or psychiatric disorder (ADHD, CD, ODD, depressive symptoms); verbal IQ > 80 (WISC-III)	fMRI task related: go/no-go task	Whole-brain patterns of activity and ROI: IPC (BA40), MFC (BA46), ACC (BA32), and striatum; two one sample *t*-test; *p-*Values statistically corrected for multiple comparisons; path model construct on structural equation modeling	Common pattern between DCD and TD: ACC (BA32), SMA (BA6), OFC (BA47), MFC (BA46), IPC (BA39&40), insula (BA13), and striatum; DCD > TD activation in left hemisphere; DCD < TD activation in right hemisphere; connectivity analysis: DCD showed stronger path coefficients in the left hemispheric network than in the right	DCD = TD for correct inhibitions; DCD > TD for omissions; go responses were slower and more variable in DCD than TD
Kashiwagi et al. (69)	12 DCD (including three ADHD, three DD, two DD + ADHD) 12 TD	10 years, 9 months (11.6 months); R = 9–12 years 10 years 5 months (11.9 months); R = 9–12 years	12M 12M	DCD: DSM-IV criteria, parent report, MABC < 15th, >3 SNS; no neurological/psychiatric disorders; FIQ > 90 (WISC-III); TD: normal intellectual development (Raven’s progressive matrices test)	fMRI task related: (1) tracking condition (track moving target by manipulating joystick); (2) watching condition (watch moving target without hand manipulation)	Whole-brain patterns of activity analyzed; *p* < 0.001 (voxel level); *p* < 0.05 (correction for multiple comparisons at the cluster level for the entire brain)	DCD = TD during (fixation) and (watching) conditions; DCD < TD activation in superior and inferior parietal lobe in the left posterior parietal cortex and left poscentral gyrus for contrast (tracking vs. watching)	DCD > TD for distance between target and cursor and change in velocity of the cursor
Zwicker et al. (70)	7 DCD 7 TD	10.8 (1.5); R = 8–12 years 10.9 (1.5); R = 8–12 years	6M, 1F 4M, 3F	DCD: MABC-2 ≤ 16th, DCDQ; TD: MABC-2 > 25th, “probably not DCD” on DCDQ; IQ > 80 (KBIT-2), no ADHD	fMRI task related: trail-tracing task (flower MABC)	Whole-brain patterns of activity analyzed using ANOVA; corrected for multiple comparisons; cor. < 0.01; cluster size *k* > 200	DCD > TD activation in left IPC (BA40); in right MFG (BA46), SG (BA40), LG (BA19), PG (BA30), PCG (BA30), PrG (BA6), STG (BA41), cerebellar Lobule VI; TD > DCD activation in left precuneus (BA39), SFG (BA8), IFG (BA47), PoG (BA2); in right STG (BA13)	DCD = TD
Zwicker et al. (71)	7 DCD 7 TD	10.8 (1.5); R = 8–12 years 10.9 (1.5); R = 8–12 years	6M, 1F 4M, 3F	DCD: MABC-2 ≤ 16th, DCDQ; TD: MABC-2 > 25th, “probably not DCD” on DCDQ; IQ > 80 (KBIT-2), No ADHD	fMRI task related: trail-tracing task (flower MABC); two scans: Day 1 and Day 5	Whole-brain patterns of activity analyzed using 2 × 2 [group (DCD, TD) × time (early practice, retention test)]; ANOVA corrected for multiple comparisons; cor. < 0.005; cluster size *k* > 200	DCD < TD activation in right IPC (BA40), LG (BA18), MFG (BA9); in left FG (BA37), IPC (BA40); in right cerebellar (crus I) and left cerebellar (Lobule VI and IX) at both early practice and retention	DCD = TD
Zwicker et al. (72)	7 DCD 9 TD	10 years, 10 months (1 year, 6 months); R = 8–12 years, 4 months 10 years, 4 months (1 year, 7 months); R = 8 year, 1 month–12 years, 6 months	6M, 1F 6M, 3F	DCD: MABC-2 ≤16th, DCDQ; IQ > 80 (KBIT-2), no ADHD	DTI: 60 slices (slice thickness = 2.2 mm; voxel size = 2.2 mm^3^); 16 independent orientations (*b* = 1,000 s/mm^2^)	Tracts analyzed: corticospinal tract, posterior thalamic radiation, and superior and middle cerebellar peduncles in MD, FA, and AD; ROI in posterior limb of internal capsule (two limiting ROI in white matter under precentral gyrus and in cerebral peduncle) and at posterior thalamus and in white matter under postcentral gyrus and in superior and middle cerebellar peduncles; analysis of covariance (covariate: age); *p* < 0.05 for all calculations; uncorrected for multiple comparison for ROI	DCD = TD in FA; DCD < TD in MD in corticospinal tract	None
Debrabant et al. (73)	17 DCD 17 TD	9.4 (0.6); R = 7–10 years (LH = 3) 9.2 (0.9); R = 7–10 years (LH = 2)	14M, 3F 14M, 3F	DCD: MABC-2 ≤5th;TD: MABC-2 > 16; IQ > 85 (WISC-III); no other diagnosed of developmental disorders (ADHD or autism), or medical condition	fMRI task related: (1) predictive visual pacing (press button when stimuli appears on screen); (2) unpredictive visual pacing (stimuli randomly presented); (3) self-pacing (control, pressing the response button)	Analysis of covariance (covariate: mean RT); *p* < 0.001; corrected for multiple comparisons; cluster size *k* > 15	Within group: TD (unpredictive > predictive): right DLPFC, MFG, and IFG; TD (predictive > unpredictive): no region; DCD (unpredictive > predictive) and (predictive > unpredictive): no region; between groups: DCD < TD activation in right DLPFC, TPJ; in left posterior cerebellum (crus I) for contrast (unpredictive > predictive); DCD = TD for contrast (predictive > unpredictive)	Between groups: significant effects for visual pacing condition (predictive, unpredictive); within groups: (predictive > unpredictive) for TD; no difference between the two for DCD
McLeod et al. (74)	7 DCD 21 ADHD 18 DCD + ADHD 23 TD	13.0 (2.5); R = 8–17 years (LH = 1) 12.5 (2.9); R = 8–17 years (LH = 2) 11.5 (3.0); R = 8–17 years (LH = 4) 11.3 (2.8); R = 8–17 years (LH = 2)	5M, 2F 20M, 1F 14M, 4F 11M, 12F	DCD: MABC-2 < 16th, DCDQ; ADHD: DSM-IV criteria; DICA-IV, or CPRS-R > 95th; no metabolic/genetic conditions, epilepsy, cerebral palsy, ID, ASD, FASD, psychiatric disorder, VLBW, or prematurity; IQ > 80 (WASI); no medication for ADHD	fMRI-rs; FC in brain regions connected with M1; T2* (5 min); fixation cross	Analysis of covariance (covariate: age); *p* < 0.05 (cluster level); cluster size *k* > 75	DCD < TD in bilateral IFG, IC, STG, caudate; in right FOC, SG, nucleus accumbens, pallidum, and putamen; DCD > DCD + ADHD in bilateral caudate, anterior STG; in left PC, PoG, FC; in right IFG, POC	None
Langevin et al. (75)	9 DCD 27 ADHD 23 DCD + ADHD 26 TD	12.2 (2.7); R = 8–17 years 11.8 (3); R = 8–17 years 11.4 (2.9); R = 8–17 years 11.6 (3.2); R = 8–17 years	7M, 2F 24M, 3F 19M, 3F 14M, 12F	DCD: MABC-2 < 16th, DCDQ; ADHD: DSM-IV criteria; DICA-IV, or CPRS-R > 95th; no metabolic/genetic conditions, VLBW, prematurity, epilepsy, cerebral palsy, ASD; IQ > 80 (WASI); no medication for ADHD	DTI: 26 axial–oblique slices (slice thickness = 4.0 mm; no interslice gaps) covering the entire brain; 11 non-linear directions (*b* = 850 s/mm^2^)	Three white matter tracts analyzed: corpus callosum, SLF, cingulum; differences in FA, MD, RD, and AD, ANOVA of DTI measures performed for each tract subdivision; *p* < 0.05	DCD < TD for FA in the left lateral SLF III; DCD = TD in MD for all tracts	None
Langevin et al. (76)	14 DCD 10 ADHD 10 DCD + ADHD 14 TD	9 years, 9 months (1 year, 7 months); R = 8–17 years (LH = 3) 9 years, 9 months (1 year, 3 months); R = 8–17 years (LH = 1) 9 years, 7 months (2 years, 3 months); R = 8–17 years (LH = 0) 11 years 9 months (3 years); R = 8–17 years (LH = 2)	5M, 9F 6M, 4F 8M, 2F 8M, 6F	DCD: MABC-2 < 16th, DCDQ; ADHD: DSM-IV criteria; DICA-IV, or CPRS-R > 95th; no metabolic/genetic conditions, VLBW, prematurity, seizure disorder, cerebral palsy, ASD; IQ > 80 (WASI); no medication for ADHD	MRI; CT; 2*3D T1 (varying inversion times: 766 and 780 ms); RT = 7.4 ms; ET = 3.1 ms; FOV = 256 mm; slice thickness = 0.8 mm; 28 cortical regions	ANOVA; *p* < 0.05; uncorrected for multiple comparisons	DCD < TD right medial orbitofrontal cortex; multiples differences in CT between DCD + ADHD and TD, DCD or ADHD alone	None
Licari et al. (77)	13 DCD 13 TD	9.6 (0.8); R = 8–10 years 9.3 (0.6); R = 8–10 years	13M 13M	DCD: MABC-2 < 5th; TD: MABC-2 > 15th; no ADHD	fMRI task related: (1) sequential finger-thumb task (touching each finger onto their thumb one at a time); (2) repetitive hand-clenching task (opening and closing their hand)	ANOVA (differences between conditions and groups); threshold of *p* < 0.05 (FDR corrected); cluster size *k* > 15	DCD < TD activations in left SFG (BA9), IFG (BA44), and DCD > TD activation in right PoG (BA3) for (finger-sequencing) condition; DCD = TD for (hand-clenching) condition	Between groups: DCD > TD for contralateral motor overflow on both tasks; within groups: finger-sequencing >hand clenching in motor overflow for DCD whereas no differences for TD
Reynolds et al. (78)	14 DCD 12 TD	10.08 (1.31); R = 7.8–11.6 years 10.10 (1.15); R = 8.33–12.00 years	14M 12M	DCD: clinician report; MABC-2 ≤ 16th, no ADHD, no ASD; TD: MABC-2 ≥ 20th	fMRI task related: (1) action observation (view finger-sequencing task without execution); (2) action execution (performed finger-sequencing task with just first hand stimulus image); (3) action imitation (viewed sequencing task and imitated actions as they observed)	ANOVA FWE corrected level of *p* < 0.05 followed by an uncorrected level of *p* < 0.001; 15 ROI: regions in pars opercularis of IFG, supplementary and premotor areas, posterior/parietal lobe, and superior temporal sulcus; Bonferroni corrected within each ROI < 0.0083	Whole brain: DCD = TD for (action execution) or (action imitation) conditions; DCD > TD activation in bilateral PrG; in right precuneus, pars opercularis of right IFG; in left MTG, PCC for (observation) condition; ROI: DCD = TD; interaction effect between group and task conditions in pars opercularis DCD < TD activation during (imitation) and DCD > TD activation during (observation)	Unreported
Debrabant et al. (79)	21 DCD 20 TD	9 years, 2 months (10 months); R = 8–10 years (LH = 3; M = 2) 9 years, 4 months (7 months); R = 8–10 years (LH = 3; M = 1)	18M, 3F 16M, 4F	DCD: MABC-2 ≤ 5th; (DCDQ/MABC-2-C); TD: MABC-2 > 16th, MABC-2-C; IQ > 85 (WISC-III); no additional clinical conditions	DTI: 60 contiguous sagittal slices (slice thickness = 2.0 mm; voxel size = 2.0 mm^3^) covering the entire brain; 15 diffusion gradients along 30 non-collinear directions (*b* = 1,400 s/mm)	Differences in FA, RD, and AD; fiber tractography combined with graph theoretical analyses to evaluate whole-brain connectomics; unreported level of significance	DTI: DCD compared with TD (1) decrease in FA and increase in RD in left retrolenticular limb of the internal capsule; (2) lower FA and higher RD in right retrolenticular limb of the internal capsule; (3) lower FA in sensorimotor tracts and altered structural connectivity; graph theorical analyses: DCD < TD in clustering coefficient, global, and local efficiency, especially, nodal efficiency at cerebellar Lobule VI and right parietal superior gyrus	None
Caeyenberghs et al. (80)	11 DCD 15 ASD 8 DCD + ASD 19 TD	8.82; R = 8–12 years 9.4; R = 8–12 years 9.75; R = 8–12 years 9.68; R = 8–12 years	11M 14M, 1F 8M 8M, 11F	DCD: DSM-IV-TR or DSM-5 criteria, MABC-2 < 15th; ASD: DSM-IV-TR or DSM-5 criteria, SRS, ADOS; TD: MABC-2 > 15th; no genetic condition, VLBW, seizure condition, cerebral palsy, neurological/psychiatric disorder, ADHD, IQ > 75 (WISC-III)	MRI; CT, 2*3D T1 (varying inversion times: 766 and 780 ms); RT = 2,250 ms; ET = 4.18 ms; FOV = 176 mm × 256 mm; slice thickness = 1 mm; 68 cortical regions	CT corrected for mean CT; area under the curve statistics; *p* < 0.05; uncorrected for multiple comparisons	DCD > TD clustering coefficient in right lateral OFC; DCD > DCD + ASD clustering coefficient of right PCG, PoG; in left transverse temporal gyrus; DCD < DCD + ASD clustering coefficient in left LG, pars opercularis of the left IFG, temporal pole; in right entorhinal cortex, MOC	None
Biotteau et al. (9)	16 DCD 16 DCD + DD 16 DD	9.6 (1.7); R = 8–12 years 9.9 (1.1); R = 8–12 years 10.3 (1.3); R = 8–12 years	12M, 4F 10M, 6F 9M, 7F	DCD: MABC-2 ≤ 5th; DD: MABC-2 > 16; French reading tests <−1.5SD, IQ > 85 (WISC-III); no other diagnosed of developmental disorders (ADHD, SLI, ASD), or medical condition	fMRI task related: (1) overtrained finger sequence tapping task; (2) unentrained finger sequence tapping task	Whole-brain; ANOVA FWE corrected level of *p* < 0.05 followed by an uncorrected level of *p* < 0.001; cluster size *k* > 50	DCD > DD activations in bilateral CG (BA31, BA24), SC (BA4, BA3), premotor cortex (BA6), TPC (BA40, BA41, BA42, BA43, BA44, BA22); in right insula (BA 13), anterior cerebellum; in left thalamus for (overtrained) condition; and higher activity in bilateral CG (BA31, BA24), thalamus; in right caudate, claustrum for (unentrained) condition; DCD > DCD + DD activation in right CG (BA24, BA31, BA32), TPC (BA7, BA21, BA22, BA31, BA37, BA41, BA42, BA43), PrG (BA4); in left premotor cortex (BA6), thalamus, globus pallidus; in right anterior and posterior cerebellum for (overtrained) condition; and higher activity in right CG (BA31, BA23) for (unentrained) condition; DCD + DD = DD	DCD = DD + DCD = DD

***Sample:** ADHD, attention-deficit hyperactivity disorder; ASD, autism spectrum disorder; CD, conduct disorder; DCD, developmental coordination disorder children; DD, developmental dyslexia; F, females; FASD, fetal alcohol spectrum Disorder; FIQ, full intelligence quotient; ID, intellectual disability; IQ, intelligence quotient; LH, left handed; M, males; ODD, opposite defiant disorder; R, range; RD, reading disabilities; SLI, specific language impairment; SNS, soft neurological signs; TD, typically developing children; VLBW, very low birth weight; **Tests:** ADOS, autism diagnostic observation schedule; CPRS-R, Conners’ Parent Rating Scale-Revised; DCDQ, developmental coordination disorder questionnaire; DICA-IV, Diagnostic Interview for Children and Adolescents-IV; DSM, diagnostic and statistical manual of mental disorders; FTT, finger-tapping task; KBIT-2, Kaufman Brief Intelligence Test, second ed; MABC, movement assessment battery for children; MABC2-C, checklist MABC-2; ROCF, Rey–Osterrieth complex figure; SRS, Social Responsiveness Scale; SRTT, serial reaction time task; WASI, Wechsler Abbreviated Scale of Intelligence; WISC, Wechsler Intelligence Scale for Children; ZNA, Zurich neuromotor assessment; **Brain acquisition and analysis:** AD, axial diffusivity; CT, cortical thickness; DTI, diffusion tensor imaging; ET, echo time; FA, fractional anisotropy; FC, functional connectivity; FDR, false discovery rate; fMRI, functional magnetic resonance imaging; fMRI-rs, functional magnetic resonance imaging-resting state; FOV, field of view; FWE, family wise error; MD, mean diffusivity; MRI, magnetic resonance imaging; RD, radial diffusivity; ROI, region of interest; RT, repetition time; **Brain areas:** ACC, anterior cingulate cortex; CG, cingulate gyrus; DLPFC, dorsolateral prefrontal cortex; FC, frontopolar cortex; FG, frontal gyrus; FOC, frontal operculum cortex; IC, insular cortices; IFG, inferior frontal gyrus; IPC, inferior parietal cortex; LG, lingual gyrus; M1, primary motor cortex; MC, motor cortex; MFC, middle frontal cortex; MFG, middle frontal gyrus; MOC, medial orbitofrontal cortex; MTG, middle temporal gyrus; OFC, orbito frontal cortex; PC, premotor cortex; PCG, posterior cingulate gyrus; PG, parahippocampal gyrus; POC, parietal operculum cortex; PoG, postcentral gyrus; PrG, precentral gyrus; SC, sensorimotor cortex SFG; superior frontal gyrus; SG, supramarginal gyrus; SLF, superior longitudinal fasciculus; SMA, supplementary motor area; STG, superior temporal gyri; TPC, temporoparietal cortex; TPJ, temporoparietal junction*.

We identified nine fMRI studies, two structural MRI studies, and three DTI studies. All the children had motor problems in daily life and, for the most part, had been assessed with the MABC (one study did not clearly report the recruitment criteria). It should be noted that the children in the comparison groups were not always assessed on motor or cognitive criteria. Four studies clearly included comorbid DCD, but comorbidities for the DCD and comparison groups were not generally explored beyond the more usual ones (i.e., autism and ADHD, never DD or SLI). The studies included only small numbers of participants, varying from 7 to 21. All of them included children aged 7–12 years (ranges: 8–12, 8–10, and 7–10), except for three studies, where mean ages varied from 8 to 17 years. A variety of tasks were performed during the fMRI scans (visuomotor, finger sequence, tracking, motor timing, go/no-go, and trail-tracing).

**Figure 1:**
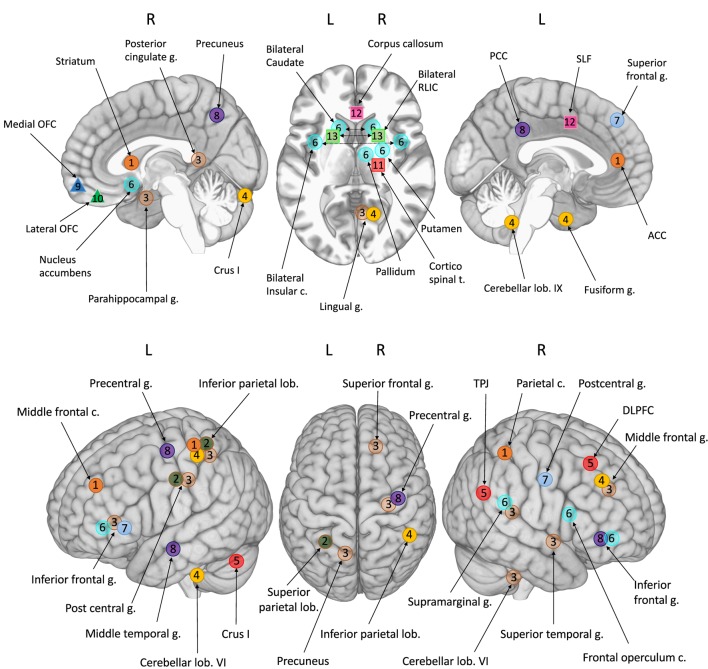
**Foci of atypical brain involvement reported from neuroimaging studies of children with DCD compared to TD children**. *Studies*: circles 1–8: fMRI; triangles 9–10: sMRI; squares 11–13: DTI. Each number/color represented one study. [1] Querne et al. (68); [2] Kashiwagi et al. (69); [3] Zwicker et al. (70); [4] Zwicker et al. (71); [5] Debrabant et al. (73); [6] McLeod et al. (74); [7] Licari et al. (77); [8] Reynolds et al. (78); [9] Langevin et al. (76); [10] Caeyenberghs et al. (80); [11] Zwicker et al. (72); [12] Langevin et al. (75); [13] Debrabant et al. (79). *Brain areas*: ACC, anterior cingulate cortex; c., cortex; DLPCF, dorsolateral prefrontal cortex; g., gyrus; lob., lobule; OFC, orbito frontal cortex; PCC, posterior cingulate cortex; RLIC, retrolenticular part of the internal capsule; SLF, superior longitudinal fasciculus; TPJ, temporoparietal junction; t, tract.

The most striking result of this review is that no two studies observed the same differences. For this reason, we provide a detailed description of each individual study in the following section. As illustrated in Figure 1, few regions are consistent across studies, owing to several different factors that are summarized below, to avoid having to reiterate them in each individual study description.

First, part of the explanation may lie in the heterogeneity of the sample. DCD is heterogeneous in nature, but this heterogeneity may be increased by (1) the use of different MRI scanners (scanning site) with specific acquisition designs, (2) the administration of different tasks during the fMRI (go/no-go, finger tapping, tracing tasks, etc.), and (3) comorbidity. Regarding this last point, it is surprising to note that the vast majority of studies failed to explore DCD comorbidities other than the most common ones. While ADHD and ASD were excluded in some studies, DD and SLI were only excluded in one of the 14 studies (9), despite being frequently associated with DCD (9, 81, 82). A fourth source of heterogeneity is the use of different assessment batteries to select the children. Although the MABC is now generally used to assess motor functions (used in 13/14 studies), cutoff scores for inclusion in the DCD and TD groups can vary. Although the 15th percentile is used to determine the clinical status of children with DCD, researchers prefer to use the 5th percentile of the motor test score to ensure that the sample is best suited for answering the research question (83). Only four of the 14 studies respected this threshold (9, 73, 77, 79). All the other studies included children below the 15th or the 16th percentile. Finally, heterogeneity may be increased by a large age range. While most of the studies included children aged 7–12 years (ranges: 8–12, 8–10, and 7–10), in three studies (74–76), mean ages varied between 8 and 17 years. It is, therefore, very difficult to directly compare the findings of these studies with those of previous ones.

Second, comparison with a control group was problematic in the majority of studies. It seems very important to ensure that the TD group has none of the signs listed in the clinical criteria for DCD (83), especially a motor performance score above the 15th percentile, but these do not always appear to have been verified. In some cases, the TD group did not even undergo a motor assessment (68, 69, 78). Additionally, we frequently found that there was no safety margin between the TD and DCD groups (e.g., DCD below the 15th percentile, TD above the 15th). Thus, fewer than half the studies (70, 71, 73, 77, 79) clearly used a motor inclusion criterion for TD children. In the remaining ones, those children who were not selected for the DCD group were included in the TD group. Additionally, the TD group was not systematically tested for IQ, or for comorbidities such as ADHD and ASD [see, for example, Reynold et al. (78)]. One study (9) did not even use control groups. These are all potential sources of bias, making comparisons difficult, if not impossible.

Third, some results need to be interpreted with the utmost caution, owing to methodological inconsistencies in inclusion. For example, the large age ranges mentioned above for McLeod et al. (74) and Langevin et al. (75, 76) are highly problematic for the intrinsic interpretation of findings. Even when analyses were performed using age as a covariate, the inclusion range was too great, given the rapid developmental maturation of the brain (84, 85). Other results also need to be interpreted with great care, owing to significant differences between the DCD and TD groups in age, sex distribution, IQ, handedness, or level of attention (71, 74–76) – all factors known to influence the brain’s structure or functioning (84–88). Furthermore, the DCD group was generally very small, making it extremely difficult to lend credence to the results (sample varying from 7 to 21 children with DCD, depending on the study; fewer than 10 in 6 studies, and fewer than 15 in 11 studies). Sometimes, even though the numbers of children differed considerably between the DCD and TD groups, tests were still conducted between the two [e.g., 7 DCD vs. 23 TD in McLeod et al. (74)]. All these factors can introduce bias into the data analysis and, by extension, undermine the authenticity of the results.

A final issue concerns the absence of methodological MRI standards. Some studies investigated the whole brain, while others were interested in a specific region. Statistical power varied from one study to another, and was sometimes uncorrected for multiple comparisons [despite 68 brain regions in Caeyenberghs et al. (80) and 28 in Langevin et al. (76)]. In some cases, the authors talked about differences even when the voxel size was very small [e.g., below 20 in Reynolds et al. and Debrabant et al. (73, 78)]. It should also be noted that studies often failed to find significant group differences for brain data and, therefore, focused on correlations between behavioral data.

### Functional MRI Studies

#### Querne et al. (68)

Using fMRI during a go/no-go task performed by children with DCD and TD, these authors assessed the impact of DCD on effective connectivity applied to a putative model of inhibition. No difference in behavioral performances was found between the DCD and TD groups (even if DCD responses were slower and more variable than TD responses). However, structural equation modeling indicated that the coefficients of the paths from both the middle frontal cortex (MFC) and ACC to the inferior parietal cortex (IPC) increased in the children with DCD, especially for the left hemisphere. Moreover, in the children with DCD, coefficients of the paths between the striatum and parietal cortex decreased in the right hemisphere. The authors suggested that DCD can be characterized by abnormal brain hemispheric specialization during development (executive functions tended to recruit a left-lateralized network in DCD and a right-lateralized network in TD). They also hypothesized that children with DCD compensate for their poor efficiency by more actively engaging the ACC, thus maintaining a good level of inhibition.

#### Kashiwagi et al. (69)

Using fMRI during a visuomotor task (visually guided tracking task), this study was designed to identify the brain mechanisms underlying clumsiness in children with DCD and TD. The behavioral performances of children with DCD were significantly less accurate than those of TD. Between-group differences showed that there was less brain activity in the superior and inferior parietal lobes, left posterior parietal cortex (PPC), and left postcentral gyrus in the children with DCD than in the TD children. The authors suggested a link between the left PPC and clumsiness in DCD.

#### Zwicker et al. (70)

Using fMRI during a fine motor trail-tracing task, the authors measured brain activation patterns in children with DCD. Similar levels of behavioral performance were noted. Between-group differences were found in patterns of brain activity. The children with DCD had greater activation in nine areas, essentially in the right hemisphere: middle frontal gyrus, supramarginal gyrus, lingual gyrus, parahippocampal gyrus, posterior cingulate gyrus, precentral gyrus, superior temporal gyrus, cerebellum (Lobule VI), and left inferior parietal lobule. They had less activation in five brain regions: left precuneus, superior frontal gyrus, IFG, postcentral gyrus, and right superior temporal gyrus/insula.

#### Zwicker et al. (71)

Using fMRI, the authors mapped brain activity associated with skilled motor practice of a trail-tracing task (5 days’ practice, two scans: days 1 and 5) in children with DCD [same population sample as in Zwicker et al. (70)]. The children with DCD did not show any improvement in motor accuracy following skilled practice. Between-group differences showed that nine brain areas were less activated in the children with DCD at both early practice and retention: the bilateral inferior parietal lobules, right lingual gyrus, right middle frontal gyrus, left fusiform gyrus, right cerebellum (Crus I), left cerebellum (Lobule VI), and left cerebellum (Lobule IX).

#### Debrabant et al. (73)

Using fMRI during a visuomotor reaction time task (sequences of visual stimuli with predictive or unpredictive interstimulus intervals, ISIs), the authors investigated the neural correlates of motor timing in DCD. The children with DCD reacted more quickly to predictive ISIs than TD children did, and there was no difference in their brain activation between the two conditions (TD exhibited greater activation in the right DLPFC and right IFG in response to unpredictive vs. predictive ISIs). Between-group differences in the predictive condition showed that the children with DCD activated their DLPFC, temporoparietal junction (TPJ), and left posterior cerebellum (Crus I) less. The authors concluded that the children with DCD needed to perform extra processing, owing to impaired predictive encoding.

#### McLeod et al. (74)

Using resting-state fMRI, the authors investigated the functional connections of the motor network in children with DCD and/or ADHD and predicted that, compared with TD, the children with DCD and/or ADHD would exhibit altered functional connectivity (FC) between the primary motor cortex (M1) and brain regions involved in motor functioning and sensorimotor processing. We excluded results for the ADHD group, given that this pathology was not the subject of our review. Between-group differences are, therefore, only shown for the DCD, DCD + ADHD, and TD groups [for details of the ADHD group, see McLeod et al (74)]. Compared with TD, the DCD group demonstrated decreased FC with M1 for the bilateral IFG, right frontal operculum cortex, right supramarginal gyrus, bilateral insular cortices and superior temporal gyri, bilateral caudate and right nucleus accumbens, pallidum, and putamen. The DCD + ADHD group exhibited lower FC with M1 for the right motor cortex, left supramarginal gyrus, bilateral postcentral gyri, left putamen, left pallidum, and left amygdala, and greater FC for the left frontopolar cortex and lingual gyrus. The children with DCD + ADHD exhibited greater FC between M1 and the bilateral caudate nuclei and anterior superior temporal gyri, left premotor cortex, postcentral gyri and frontopolar cortex, and right IFG and parietal operculum cortex. The authors concluded that the decreased FC between M1 and the striatum and angular gyrus (observed in all the groups) indicated that these brain regions are common neurophysiological substrates underlying both DCD and ADHD. They also suggested that the co-occurrence of neurodevelopmental disorders may have a distinct impact on FC (unique alterations in FC between M1 and sensory networks for these children).

#### Licari et al. (77)

Using fMRI during two tasks (finger sequencing and hand clenching), the authors investigated cortical activation patterns contributing to increased motor overflow in children with DCD. Behavioral results showed that the children with DCD performed more poorly than TD on both tasks. Analysis failed to reveal between-group differences in the hand-clenching condition. However, compared with TD, the children with DCD activated their left superior frontal gyrus (BA 9) and left IFG (BA 44), and right postcentral gyrus (BA 3) more in the finger-sequencing task.

#### Reynolds et al. (78)

Cortical activations during the performance of an imitative finger-sequencing task (observation, action execution, and action imitation) were observed through the use of fMRI. The authors hypothesized a deficit in the mirror neuron system (MNS). A between-group analysis revealed differences in six areas (right IFG, precentral gyrus, precuneus, left precentral gyrus, middle temporal gyrus, and posterior cingulate), but only in the observation condition. Region of interest analysis revealed less activation in the pars opercularis in the DCD group in the imitation condition. The authors concluded that the results supported a possible MNS dysfunction.

#### Biotteau et al. (9)

Using fMRI during a finger-tapping task administered to children with DCD with or without comorbidity, the authors assessed the impact of comorbidity in DCD on brain functioning. No difference was found in behavioral performances between DCD and the other groups. However, compared with the DD group, results revealed greater activity in the bilateral cingulate gyrus (BAs 24 and 31), bilateral sensorimotor cortex (BAs 3 and 4), bilateral premotor cortex (BA 6), bilateral temporoparietal cortex, right insula (BA 13), left thalamus and left globus pallidus, right caudate and right claustrum, and right anterior and posterior cerebellum. The authors suggested that, compared with the DD and DD + DCD groups, the DCD group was characterized by a distinct pattern of functioning in the neural correlates recruited for procedural learning. The DD and comorbid groups were very close, whereas the DCD group presented specific characteristics, raising the issue of the nature of motor problems in DD.

### Structural MRI Studies

#### Langevin et al. (76)

Using structural MRI, the authors examined whether comorbid motor and attention problems influence cortical thickness in children. They compared the brain patterns of four groups. Between-group differences in cortical thickness were large, but in summary the children with DCD + ADHD had a greater overall decrease in cortical thickness than the children with DCD or ADHD alone (concentrated in the frontal, parietal, and temporal lobes). The children with DCD alone only differed from TD on the right medial orbitofrontal cortex (MOC), which was thinner in DCD. The authors concluded that the DCD + ADHD co-occurrence is associated with a distinct overall pattern of decreased regional cortical thickness, highlighting the unique neurobiology of comorbid neurodevelopmental disorders.

#### Caeyenberghs et al. (80)

Using structural MRI, this study aimed to address the question of whether abnormal connectivity in DCD overlaps with that seen in ASD or comorbid DCD + ASD. The authors investigated differences in the global and regional topological properties of structural brain networks (small-world networks between 68 brain regions, based on cortical thickness) in 53 children divided in four groups. Between-group differences between ASD (with or without DCD) and other groups were large, but are not provided here (see Caeyenberghs et al. (80) for details). The DCD group exhibited only one difference from the TD group, in the right lateral orbitofrontal cortex (higher clustering coefficient). Regarding comorbidity, compared with the DCD group, the children with DCD + ASD had higher nodal clustering coefficients in the left lingual gyrus, pars opercularis of the left IFG, left temporal pole, right entorhinal cortex, and right MOC. By contrast, the DCD + ASD group had lower clustering coefficients in the right posterior cingulate gyrus, right postcentral gyrus, and left transverse temporal gyrus. The authors concluded that the DCD, ASD, and TD groups had prominent small-world properties in their cortical thickness networks, even if the overall organization of networks in the children with DCD was relatively intact, as shown by the absence of group effects on overall network parameters (global network values close to those of TD).

### DTI Studies

#### Zwicker et al. (72)

The authors measured diffusivity and fractional anisotropy (FA) in the corticospinal tract, posterior thalamic radiation, and superior and middle cerebellar peduncles in DCD. Mean diffusivity of the corticospinal tract and posterior thalamic radiation was lower in DCD than in TD. By contrast, FA in these tracts and diffusion parameters in the cerebellar pathways did not differ between groups. The authors concluded that reduced axial diffusivity in motor and sensory tracts may be implicated in DCD.

#### Langevin et al. (75)

Using DTI in children with DCD, the authors examined the three major white-matter tracts involved in attention and motor processes [corpus callosum, cingulum, and superior longitudinal fasciculus (SLF)]. They explored associations between attention/executive and motor measures, and white-matter microstructure. Results for the ADHD group are not mentioned here (see Langevin et al. (75) for details). The authors found microstructural abnormalities in the white-matter connections underlying the primary and somatosensory motor cortices that were unique to the DCD group, with FA reductions in regions of the corpus callosum underlying parietal brain regions (superior/posterior parietal cortex, corpus callosum), as well as the left SLF. Children with comorbid DCD + ADHD exhibited alterations found in children with DCD or ADHD only (two distinct callosal regions). The authors, therefore, suggested that ADHD and DCD share a common basis in the callosal structure (reflecting a neurobiological basis for motor and attention disorders in children), with regionally and functionally distinct alterations.

#### Debrabant et al. (79)

Using DTI, the authors investigated whole-brain structural connectomics to identify abnormal microstructural properties of specific sensorimotor white-matter tracts in children with DCD. First, the children with DCD displayed a significant decrease in mean FA, together with an increase in mean radial diffusivity of the left retrolenticular limb of the internal capsule. Second, DCD-related FA reductions in the left retrolenticular limb of the internal capsule were associated with poor visuomotor tracing outcomes. Third, nodal efficiency in the cerebellum (Lobule VI) and right parietal superior gyrus was found to be a significant predictor of DCD.

## Discussion

Neuroimaging studies have been conducted with a view to supporting the most promising hypotheses formulated on the basis of neuropsychological and behavioral observations in children with DCD. These point to impaired activation in the cerebellum, parietal lobes, and BG. In the following subsections, we discuss the available neuroimaging evidence.

### Cerebellum

The cerebellum was targeted in these studies because of its role in movement, balance, coordination, learning, and automatization. Four studies (70, 71, 73, 79) observed specific features within this area. Lobule VI was highlighted in three of them (70, 71, 79), and Debrabant et al. (79) concluded that it is a significant predictor for DCD. Crus I was also mentioned by Zwicker et al. (71) and Debrabant et al. (73), as well as Lobule IX (71). Our review, therefore, highlights a particular role of Lobule VI in the neuropathology of DCD. However, there is converging evidence in the literature that the cerebellum is a common source of neuropathology in children with neurodevelopmental disorders (89). Thus, although DCD is probably linked to cerebellar dysfunction, it would be difficult to use the latter as a specific signature of DCD, given the evidence of cerebellar involvement in all other neurodevelopmental disorders (e.g., ADHD, ASD, DD).

### Basal Ganglia

On account of their primary role in movement initiation, planning, motor control, learning, and automatization, the BGs are viewed as being potentially implicated in DCD. Several neuroimaging studies seem to corroborate this hypothesis (68, 72, 74, 79). Querne et al. (68) found atypical (decreased) coefficients for the paths between the striatum and parietal cortex in the right hemisphere. McLeod et al. (74) showed atypical recruitment of the caudate, nucleus accumbens, pallidum, and putamen for both DCD and DCD + ADHD. These authors even concluded that the striatum could be a common neurophysiological substrate of DCD and ADHD. Finally, using DTI techniques, Debrabant et al. (79) and Zwicker et al. (72) found particularities that mainly involved the thalamus: the corticospinal tract and posterior thalamic for Zwicker et al. (72), and the retrolenticular limb of the internal capsule for Debrabant et al. (79). Once again, however, it would be presumptuous to regard the BG as a unique brain characteristic of DCD, because of their suspected or proven involvement in a large number of neurodevelopmental disorders (90–94).

### Parietal Lobe

The parietal lobe is a promising structure in the search for neural correlates in DCD. Eleven of the 14 neuroimaging studies mentioned its involvement in DCD. Differences were found in the IPC (68–71), superior parietal cortex (69, 79), PPC (69), postcentral gyrus (69, 70, 74, 77), supramarginal gyrus (70, 74), TPJ (73), and parietal operculum cortex (74). Additionally, Querne et al. (68) noted decreased coefficients for the paths between the striatum and parietal cortex in the right hemisphere in children with DCD. These findings were consistent with those of McLeod et al. (74) who found decreased FC between M1 and the striatum and angular gyrus in the DCD group. It should, however, be noted that the authors concluded that these brain regions are common neurophysiological substrates of both DCD and ADHD, as this specific decrease was observed in all three groups. Once again, therefore, abnormalities in this brain region are probably not peculiar to DCD and could be common to other neurodevelopmental disorders.

### Other Areas

#### Limbic Lobe

There are few hypotheses of limbic lobe involvement in the physiopathology of DCD, even though several neuroimaging studies have found evidence of such involvement (68, 70, 74, 78). In particular, fMRI studies have reported differences in the ACC (68) and posterior cingulate cortex (70, 78), parahippocampal gyrus (70), insular cortices and insula (70, 74), and left amygdala (74). However, as suggested by Querne et al. (68), children with DCD may compensate for their poor efficiency by more actively engaging the cingulate cortex, thereby maintaining a good level of inhibition. This assumption can be applied to the other limbic areas mentioned.

#### Frontal Lobe

Frontal lobe dysfunction has been found in almost all MRI studies, but it is very difficult to draw the right conclusions about its involvement in DCD, owing to its unspecific nature. However, several features in specific frontal areas may appear to be potential neural correlate of DCD. First, for DCD alone compared with TD, Langevin et al. (76) noted only one difference – in the right MOC (thinner in DCD). Exactly the same result was reported by Caeyenberghs et al. (80). Using an innovative design (separate structural correlation networks based on cortical thickness), they highlighted the specific involvement of the right orbitofrontal cortex in DCD. Connectivity of the orbitofrontal cortex mainly concerned limbic areas (in particular, the insular cortex, parahippocampal regions, and amygdala, which we just mentioned as atypical in DCD) and the striatum. The MOC is also connected to the DLPFC, which was found to be implicated in the physiopathology of DCD by Debrabant et al. (73), especially, the right DLPFC. Given its role in executive functions and cognitive processes, the DLPFC seems to be an excellent signature of DCD. It should be noted that the DLPFC is connected to a variety of subcortical structures, which once again include the thalamus and BG (specifically the caudate nucleus). All in all, the DLPFC, MOC, and their connections to limbic and subcortical structures, could be viewed as solid cerebral correlates of DCD.

#### Lingual Gyrus

Differences have been noted in the lingual gyrus (70, 71, 74). This brain region is assumed to be engaged by low-level visual processes (95, 96) and to play an important role in vision, especially related to letters. The recent review by Richlan et al. (97) identified it as a specific brain characteristic for dyslexia. Comorbidity with dyslexia, which has not so far been explored in DCD neuroimaging studies, despite its frequency (98), is one possible (but speculative) explanation. The same question applies to other areas, such as the left fusiform gyrus mentioned by Zwicker et al. (71), temporal areas where differences between DCD and TD have been found in many studies (71, 74, 75, 78), and even Lobule VI (70, 71, 79), which has been designated as linked to reading-related activity (99). This raises the underlying question of how to assess all possible comorbidities when studies are performed with DCD children in order to determine what is specifically related to DCD and what is related to another comorbid disorder.

#### Abnormal Brain Hemispheric Specialization

Two studies support the notion of abnormalities in cerebral hemispheres, congruent with the older hemispheric disconnection approach (15). Querne et al. (68) evoked abnormal brain hemispheric specialization during development. These authors found that executive functions tended to recruit a left-lateralized network in DCD and a right-lateralized network in TD. Alternative findings suggest that DCD may reflect a hemispheric disconnection syndrome. In particular, Langevin et al. (75) observed reduced connectivity between the parietal region of the corpus callosum and parietal areas, compared with TD.

## Conclusion

The findings described above open up promising avenues in the quest for a possible cerebral signature for DCD.

First, the cerebellum and BG are unquestionably linked to DCD. But there is converging evidence that both are involved in several neurodevelopmental disorders and are probably indicative of the nature of neurodevelopmental disorders in general, rather than the unique and intrinsic nature of DCD. However, the cerebellum and BG are nowadays most often regarded as a single block (in a relatively general and imprecise manner). If they had to be investigated in greater detail, links might probably emerge between specific disorders and specific cerebellar or BG areas. Therefore, their degree of involvement and the precise role they play in the neuropathology of neurodevelopmental disorders have yet to be clearly defined.

Second, similar doubts can be expressed about the parietal lobe, even if its specific implication in DCD is more probable.

Third, two regions (MOC and DLPFC) closely connected to the cerebellum and BG, and also closely interconnected, could constitute a good signature of DCD.

Fourth and last, the limbic lobe, especially the cingulate cortex and parahippocampal gyrus, would appear to be viewed as compensatory brain mechanisms in DCD, more actively engaged to ensure that a good level of achievement is maintained.

Altogether, can the results of these 14 neuroimaging studies really be said to point to a neural signature for DCD? If we include the cerebellum, BG, parietal lobe, MOC, and DPFC, the answer is probably yes, but uncertain. Indeed, during our review of these neuroimaging studies, it became clear that the current literature struggles to find a consistent picture on neural correlates linked to DCD (Table 1; Figure 1), which prevents us to reach firm conclusions on a cerebral signature of DCD. There are many possible explanations for this.

First, there have been too few MRI studies to date, and there has been no uniformization of the tasks performed during fMRI. The use of different designs (task, paradigm, and duration), especially in a pediatric population, is a major limitation, making comparisons impossible and findings hard to interpret when it comes to determining what is related to the neurophysiological basis of DCD, to task difficulty or to compensatory mechanisms.

Second, samples have often been very small, and above all, non-homogeneous. They differed across our 14 studies for many reasons. Regarding inclusion ages, studies adhered closely to the minimum age for DCD diagnosis, only including participants aged 5 years or older, in accordance with the European Academy for Childhood Disability [EACD (100)]. However, the age range extended from 7 to 17 years which, given the development of the brain across childhood, adolescence, and puberty (85, 101), prevented researchers from extending the significance of their findings. Inclusion criteria were also a real problem. While the use of MABC (or MABC-2) received a broad consensus (13/14 studies used it), the cutoff scores used to assess children with DCD were not homogeneous. Concerning the inclusion criteria for TD, motor and/or cognitive abilities were not systematically controlled for, and even where this was done, a safety margin was not always created between the TD and DCD groups for motor skills.

Third, for the most part, the authors chose not to look for comorbidities other than the more usual ones (ASD or ADHD). Multicomorbidities in neurodevelopmental disorders are, however, extremely frequent (102), resulting in the co-occurence of two and even three or more disorders, with a high level of co-occurrence with SLI or DD for DCD (44, 81, 82). More homogeneous subtypes of DCD, excluding not only ASD and ADHD but also DD and SLI, are required, in order to distinguish the brain characteristics of DCD alone from those of other comorbid disorders. Additionally, given the absence of homogeneity in DCD, it would appear appropriate to explore the heterogeneous presentation of the children who are studied (where do they experience difficulty: in motor learning, and/or motor skill acquisition, and/or gross motor skills, and/or fine motor skills?). This has not been the case up to now, even though it could have enhanced the analysis and comprehension of the findings.

Fourth and last, can we really talk about differences when cluster sizes are below 50 or when results are uncorrected for multiple comparisons? More rigorous analysis of imaging data will be required to ensure the validity of such differences.

Thus, to confirm our conclusions about the involvement of the cerebellum, BG, parietal lobe, MOC, and DPFC in DCD, new neuroimaging studies need to be designed. Based on this review, we strongly encourage researchers:

1. to include groups more homogeneous with strict inclusion criteria for DCD and TD, especially concerning cognitive and motor tests (safety margin between the two groups is for example required), featuring larger samples;
2. to take comorbidities into account and not only autism or ADHD but also SLI or DD;
3. to pay careful attention to rigorous analysis of imaging data;
4. to use preferentially more advanced neuroimaging techniques as DTI and resting state or even to realize longitudinal structural neuroimaging studies in order to isolate cortical alterations inherent to DCD or reflecting environmental influence (e.g., poor motor experience);
5. finally, to combine several neuroimaging markers as it has already done in other pathologies (103).

Even if we know the difficulties encountered by such recommendations, it seems necessary in order to allow for the comparison of results from many studies and providing an opportunity to share imagery data.

## Author Contributions

MBi, J-MA, and YC all contributed to the elaboration of the ideas developed in the manuscript. MBi and J-MA wrote the first draft of the manuscript, table, and figure. YC completed parts of the manuscript, revised, and rewrote sections of the initial article. JT and MBl made critical amendments and essential feedback to this second version of the manuscript. PP provided critical feedback to figure and table, reorganized, and rewrote parts of the table. All authors read and approved the final manuscript.

## Conflict of Interest Statement

The authors declare that the research was conducted in the absence of any commercial or financial relationships that could be construed as a potential conflict of interest.
